# Feline strongyloidiasis: An insight into its global prevalence and transmission cycle

**DOI:** 10.1016/j.onehlt.2024.100842

**Published:** 2024-06-20

**Authors:** Huan Zhao, Richard Stewart Bradbury

**Affiliations:** College of Public Health, Medical & Veterinary Sciences, James Cook University, Townsville, QLD 4811, Australia

**Keywords:** *Strongyloides*, Strongyloidiasis, Cats, Feline, Prevalence, Transmission

## Abstract

The potential cross-transmission of *Strongyloides stercoralis* between dogs and humans has become an increasing focus of strongyloidiasis research and control programs. However, the role of cats and wild felids in the maintenance and transmission cycles of human and canine strongyloidiasis has received sparse attention. Feline strongyloidiasis epidemiology remain enigmatic. We conducted a systematic review and meta-analysis to assess the global prevalence of *Strongyloides* spp. in felines and reviewed cross-species infection studies to elucidate the transmission cycle of some feline *Strongyloides* species. Literature searched from seven databases identified 42 eligible prevalence studies published between 1985 and 2024. Of these, 44 datasets from 40 studies were included in the meta-analysis. Using a random effect model combined with the Rogan-Gladen method, we estimated the pooled global prevalence of *Strongyloides* spp. in felines at 13.3% (95% CI: 8.3–18.3%), with rates of 12.2% (95% CI: 6.7–17.8%) in domestic cats (*Felis catus*) and 20.0% (95% CI: 14.9–25.2%) in wild felids. Feline strongyloidiasis was distributed across all six WHO regions, with Africa (49.7%; 95% CI: 40.0–59.3%) and the Western Pacific (46.9%; 95% CI: 42.6–51.1%) showing the highest pooled prevalence. Subgroup analysis revealed a significantly higher prevalence of *Strongyloides* infection in stray domestic cats (29.2%; 95% CI: 6.3–52.1%) compared to pet cats (9.3%; 95% CI: 3.7–14.9) and shelter cats (4.4; 95% CI: 0–9.0). Historical cross-species transmission studies demonstrated variable susceptibility of cats to human- or canine-derived *S. stercoralis*. It remains inconclusive whether cats act as a reservoir for *S. stercoralis* infection in humans or vice versa. Feline strongyloidiasis is a prevalent condition in wild, stray, pet and shelter cats. Much of the available prevalence data does not discriminate to species level, and the role of cross-species transmission in feline *S. stercoralis* infections remains obscure. Future studies would benefit from utilising molecular genotyping tools to enable species-level phylogenetic differentiation.

## Highlights

• The global prevalence of feline strongyloidiasis was 13.3% (95% CI 8.3–18.3%).
• Feline strongyloidiasis was distributed across all six WHO regions.
• Stray cats had significantly higher *Strongyloides* prevalence than pet/shelter cats.
• Cats were poorly susceptible to human- or canine-derived *Strongyloides stercoralis*.

## 1 Introduction

*Strongyloides* (order Rhabditida, family Strongyloididae) is a genus of soil-transmitted helminths infecting a variety of terrestrial vertebrates, including humans (*Homo sapiens*) and two major companion animals of humans, dogs (*Canis lupus familiaris*) and cats (*F. catus*) [1,2]. This parasite has a unique lifecycle, characterised by alternating parasitic and free-living developmental phases [3,4]. The obligate female-only parasitic generation reproduces parthenogenetically within the host intestine. Depending on the species, eggs or hatched rhabditiform larvae (L1) are passed into the environment where they develop further into infective third-stage larvae (iL3s) (homogonic route), or into facultative dioecious free-living adults which undergo sexual reproduction to produce a new generation of iL3s (heterogonic route). The resulting iL3s then invade the host percutaneously and migrate either directly or via the pulmonary route to the intestinal mucosa, maturing into parthenogenetic adult females [3,4].

Strongyloidiasis in humans and dogs is predominantly caused by *Strongyloides stercoralis* [5]. In immunocompetent persons and dogs, *S. stercoralis* infection typically manifests as an uncomplicated yet remarkably chronic disease [6,7]. However, in cases of immunosuppression, a potentially fatal disseminated disease may ensue due to the parasite's accelerated autoinfective cycle [8]. Globally, strongyloidiasis disproportionately impacts dogs and humans living in underserved settings, with an estimate 8.1% (95% CI: 4.2–12.4%) of people [9] and 6% (95% CI: 4–8%; 868/20,627) of dogs [10] affected.

*Strongyloides* in cats remains significantly understudied, with its prevalence, transmission dynamics, and public health impact largely unknown. It has been indicated that four species of *Strongyloides* infect felines, these being *Strongyloides felis* [11], *Strongyloides planiceps* [12], *Strongyloides tumefaciens* [13] and *S. stercoralis* [14]. Contention over the taxonomy of some species persists, despite new insights provided in the molecular-genetic era.

The first *Strongyloides* species identified in cats was *S. felis* by Chandler [11] in India. On morphological grounds, Chandler [11] did not exclude the possibility of it being a subspecies of *S. stercoralis.* Since this initial discovery, *S. felis* has only been reported twice globally [15,16]. Although genotyping data for this species are unavailable, phylogenetic analyses of putative *S. felis* isolates from Thailand [16] and Myanmar [17], utilising the partial *18S rRNA* gene [16] and protein-coding mitochondrial genome [17], respectively, support it being a distinct but evolutionarily closely related species to *S. stercoralis* of both human and dog origins.

*Strongyloides planiceps* was originally discovered by Leiper in rusty tiger cats (*Prionailurus planiceps*) from Malaysia [18]. Rogers [12] subsequently described this species in domestic cats, albeit misclassifying it as a new species “*Strongyloides cati”*. *Strongyloides planiceps* is distinguishable from other feline *Strongyloides* spp. by the passage of eggs, rather than L1 larvae, in faeces [12]. Genotyping research based on partial mitochondrial cytochrome *c* oxidase subunit I (*cox 1*) gene indicated that *S. planiceps* shares a common ancestor with (human and canine derived) *S. stercoralis* [17,19]. While *S. planiceps* is believed to predominantly occur in wild felines and canines, infrequent reports of this species in domestic cats exist [[20], [21], [22]].

*Strongyloides tumefaciens* was first described by Price and Dikmans [13] in two domestic cats from the southeastern United States of America (USA). This species was designated based on characteristic colonic nodules observed in the infected cats upon necropsy [13]. Complete morphological data for this parasite are unavailable and no molecular characterisation has been attempted.

Recently, Wulcan and colleagues [23] observed similar colonic lesions in *S. stercoralis*-infected cats from St. Kitts. Morphologically, the recovered parasitic female of *S. stercoralis* resembled those described for *S. tumefaciens* by Price and Dikmans [13]. Phylogenetically, *S. stercoralis* isolates from St. Kitts cats [23] clustered closely on the *cox1* (522 bp) locus with human *S. stercoralis* isolates from Lao [24] and dog isolates from Japan [25,26] and the USA [27]. This study challenged the taxonomic validity of *S. tumefaciens*. Although numerous reports of *S. tumefaciens* [[28], [29], [30], [31]] and *S. stercoralis* infections in cats exist, none of the studies detailed how species were confirmed. Wulcan et al. [23]’s work represents the first unequivocal documentation of natural *S. stercoralis* infection in cats.

The role of companion animals in the transmission cycle of human strongyloidiasis remains enigmatic. While much research effort in this regard has been directed towards dogs [2], cats have received sparse attention. Genetic evidence thus far suggests that at least some cat-derived populations of *S. stercoralis* are potentially zoonotic [23]. It is unknown whether *S. stercoralis* or other *Strongyloides* spp. from cats are naturally transmissible to humans, or vice versa. Understanding the role, if any, cats and wild felids play in the transmission and maintenance of strongyloidiasis in both humans and dogs holds significant public health implications. Essentially, within a One-Health context, it may inform whether co-treatment of companion cats is necessary for controlling human and canine infections in endemic communities. In the hope of inspiring more research in this area, we synthesised and reviewed experimental evidence on cross-species transmission of feline *Strongyloides* species.

Currently available epidemiological data on feline strongyloidiasis are limited and disparate, with the global prevalence and distribution poorly understood. We hereby conducted the first systematic review and meta-analysis of *Strongyloides* prevalence in feline populations worldwide.

## 2 Material and methods

### 2.1 Meta-analysis of feline strongyloidiasis prevalence

#### 2.1.1 Search strategy and selection criteria

This review followed the Predefined Protocol Items for Systematic Reviews and Meta-Analyses (PRISMA) guideline. Literature search was performed within seven English language databases, including Web of Science, Scopus, PubMed, Embase, Medline, Global Health, and CINAHL. Grey literature was identified through a Google Scholar search and citation searching. The key search terms used were: (Strongyloides OR gastrointestinal helminth OR intestinal parasit* OR endoparasit*) AND (cat OR kitten OR feline OR felids). The search was not limited by language, and the publication timeframe spanned from January 1983 to January 2024.

Inclusion criteria were: 1) Peer-reviewed original research articles; 2) Studies utilising case-control, cohort, or cross-sectional study designs; 3) Articles reporting the prevalence of *Strongyloides* spp. in felines. Excluded from the review were experimental studies, review articles, case reports, case series, conference proceedings, as well as letters or correspondences.

#### 2.1.2 Data extraction and quality assessment

Two researchers conducted article screening and study selection independently. Data from the included studies were systematically organised into the following categories: authors and publication year, country of the study, host species, host type, specimen examined, diagnostic method employed, diagnostic stage, sample size, number of positive samples, prevalence (%), and identified *Strongyloides* species. The Joanna Briggs Institute Prevalence Critical Appraisal Tool, conprisng eight items, was used to assess the methodological quality and risk of bias in the included articles (Supplementary File 1).

#### 2.1.3 Statistical analysis

To ensure accurate prevalence estimations, all prevalence data underwent adjustments to accommodate the imperfect sensitivity and specificity of diagnostic tests. True Prevalence (TP) estimates were calculated using the Rogan and Gladen [32] method, as previously described [9,33]. Sensitivity and specificity data for each diagnostic test were extracted from the existing literature (Table 1, Table 2). When studies reported the presence of larvae-shedding *Strongyloides* spp. in felines, TP calculations relied on sensitivity and specificity data specific to the detection of *S. stercoralis* larvae (Table 1). In cases where *Strongyloides* eggs were identified, potentially representing *S. planiceps*, prevalence rates were adjusted using relevant diagnostic performance data for *Strongyloides* egg detection in dietarily comparable hosts, such as primates (Table 2). When multiple diagnostic techniques were employed to assess *Strongyloides* prevalence, the method with the highest sensitivity was chosen during the calculation of the pooled prevalence to prevent potential overestimation.

**Table 1:** Sensitivity and specificity of different diagnostic techniques for *Strongyloides stercoralis* larvae detection (reference standards of the reviewed studies were faecal-based techniques only).

	Sensitivity (%)	Specificity (%)	References
Agar plate culture	89	100	[34,35]
Baermann technique	72	100	[34,35]
Formalin-ether/ethyl acetate sedimentation	48	100	[34,35]
Spontaneous sedimentation	27	100	[36]
Direct smear	18	100	[34]
Faecal flotation^a^	3	100	[37]
FLOTAC	5	100	[38]
Necropsy	99⁎	100	No data

⁎ Estimate based on expert opinion in the absence of any available published data;

a Based on the Willis saturated solution passive flotation method.

**Table 2:** Sensitivity and specificity of different diagnostic techniques for *Strongyloides* spp. egg detection (reference standards of the reviewed studies were faecal-based techniques only).

	Host	Sensitivity (%)	Specificity (%)	References
Spontaneous sedimentation	*Mandrillus sphinx*	49	100	[39]
McMaster	*Mandrillus sphinx*	88	100	[39]
Faecal flotation	*Mandrillus sphinx*	88⁎	100	No data

⁎ Extrapolated from the sensitivity of the methodologically comparable faecal flotation method for *Strongyloides* egg detection in *Mandrillus sphinx*, as no published data were available.

Pooled prevalence estimates were calculated using the random effects model, employing the inverse variance method for weighting, and reported with a 95% confidence interval (CI). Heterogeneity among studies was evaluated using the Cochran Q test and the inconsistency index (*I*^2^), with *I*^2^ values exceeding 75% indicating high heterogeneity. Subgroup analysis was conducted based on several variables including World Health Organisation (WHO) regions, host species, host types (pet, stray, shelter, and wild), specimen types (faeces or gastrointestinal contents), and *Strongyloides* species. All statistical analyses were performed using R studio 4.2.0, with a significance level defined as *p* < 0.05.

### 2.2 Review of cross-infection studies

A literature search was conducted in PubMed and Google Scholar up to January 2024, using the terms “*Strongyloides*” AND experiment*. There were no language, publication type, or time restrictions. Peer-reviewed original studies reporting experimental cross-species transmission of feline *Strongyloides* species were eligible for inclusion. Review articles, conference proceedings, and correspondence were excluded. Citation searching was employed to identify grey literature. Data from the included studies were extracted based on year(s) of the study, geographic origin of infection, *Strongyloides* species involved, experimental hosts, mode and intensity of inoculation, diagnostic methods, and prepatent and patent periods of infection. No statistical analysis was performed on the data.

## 3 Results

### 3.1 Meta-analysis of feline strongyloidiasis prevalence

#### 3.1.1 Overview of the studies

**Fig. 1:**
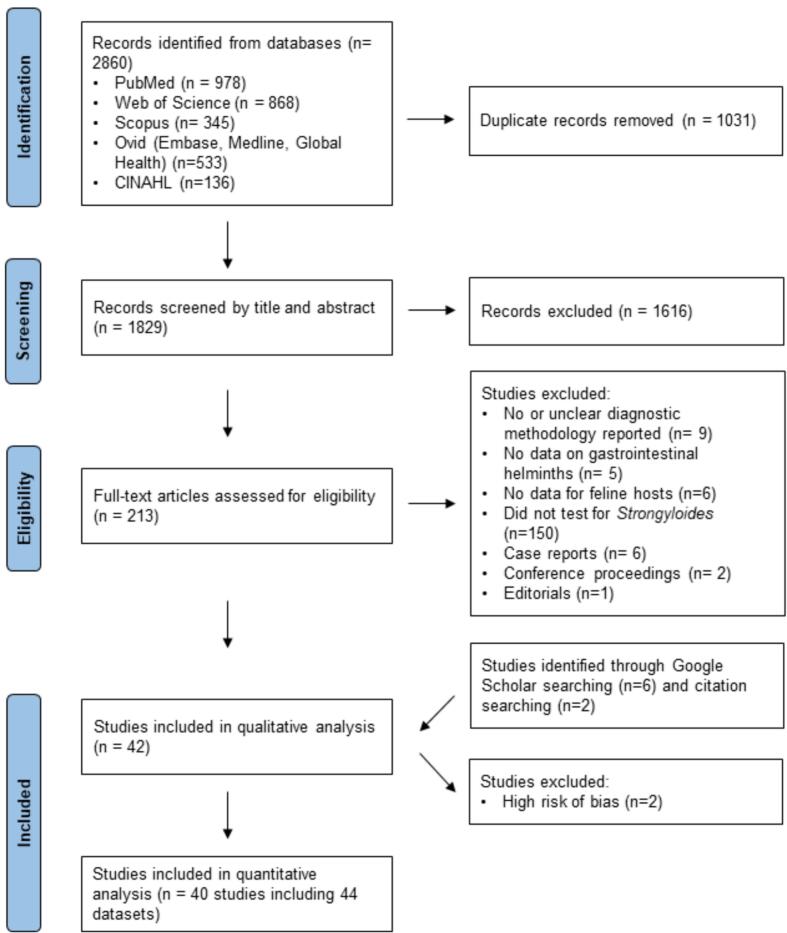
Predefined Protocol Items for Systematic Reviews and Meta-Analyses (PRISMA) flow diagram of the search strategy.

A total of 42 studies were included in the review (Table 3; Supplementary File 2). Quality assessment using the eight-item JBI tool revealed that the majority (35/42) demonstrated high methodological quality with a low risk of bias, scoring between 6 and 8 (Supplementary File 1). However, two studies were excluded from the quantitative meta-analysis due to methodological incompleteness and bias. One of the studies [40] examined *Strongyloides* spp. prevalence in captive wild felids in a zoo, but its small sample size (*n* = 9) limited its representativeness for the broader host population in the region. The other study [41] lacked sufficient details on sampling and diagnostic methodologies to permit meta-analysis. Consequently, 44 datasets from 40 studies were included in the meta-analysis for pooled *Strongyloides* prevalence in felines (Fig. 1, Table 3).

**Table 3:** Main characteristics of studies included in the systematic review.

Authors (year)	Country	Host species	Host type	Specimen	Diagnostic method	Stages detected	Sample size	Positive samples	Prevalence (%)	*Strongyloidiasis* species
Susilowati (1985)	Indonesia	*Felis catus*	NA	Faeces	DS, SS, FF	Eggs	192	6	3.1%	*Strongyloides* spp.
Ogassawara et al. (1986)	Brazil	*Felis catus*	Pet cats	GI	AWM	NA	54	3	5.6%	*Strongyloides* spp.
Speare & Tinsley (1987)	Australia	*Felis catus*	Pet and stray cats	Faeces	BT	Larvae	504	169	33.5%	*Strongyloides felis*
Heidt et al. (1988)	USA	*Felis rufus*	Wild felids	Faeces	FF	Eggs	8	2	25.0%	*Strongyloides* spp.
Foster et al. (2006)	USA	*Puma concolor*	Wild felids	GI	AWM	NA	18	4	22.0%	*Strongyloides* spp.
Abu-Madi et al. (2007)	Qatar	*Felis catus*	Stray cats	Faeces	FES	Larvae	824	152	18.4%	*Strongyloides stercoralis*^b^
Mekaru et al. (2007)	USA	*Felis catus*	Shelter cats	Faeces	FF	NA	344	1	0.3%	*Strongyloides stercoralis*^b^
Adams et al. (2008)	Australia	*Felis catus*	Stray cats	Faeces	FF	Eggs	28	13	46.4%	*Strongyloides* spp.
Mircean, Titilincu, & Vasile (2010)	Romania	*Felis catus*	Pet cats	Faeces	FF	NA	414	14	3.4%	*Strongyloides* spp.
Borkataki et al. (2013)	India	*Felis catus*	Stray cats	Faeces	FF, SS, McMaster	Eggs	100	28	28.0%	*Strongyloides* spp.
Mohd Zain et al. (2013)	Malaysia	*Felis catus*	Stray cats	GI	AWM	NA	543	6	1.1%	*Strongyloides* spp.
Aranda R. et al. (2013)^a^	Peru	*Panthera onca*	Wild felids captive in zoo	Faeces	DS, SS, FF	Larvae	9	2	22.2%	*Strongyloides* spp.
Aranda R. et al. (2013)^a^	Peru	*Puma concolor*	Wild felids captive in zoo	Faeces	DS, SS, FF	Larvae	4	2	50.0%	*Strongyloides* spp.
Aranda R. et al. (2013) ^Ϯ^	Peru	*Leopardus pardalis*	Wild felids captive in zoo	Faeces	DS, SS, FF	Larvae	2	2	100.0%	*Strongyloides* spp.
Aranda R. et al. (2013)^a^	Peru	*Leopardus wiedii*	Wild felids captive in zoo	Faeces	DS, SS, FF	NA	2	0	0.0%	
Riggio et al. (2013)	Italy	*Felis catus*	Pet cats	Faeces	FF, BT	NA	81	0	0.0%	
Rojekittikhun et al. (2014)	Thailand	*Felis catus*	Shelter cats	Faeces	FES	Larvae	300	2	0.7%	*Strongyloides* spp.
de Sousa et al. (2014)	Brazil	*Felis catus*	Stray cats	Faeces	SS	Eggs	12	5	41.7%	*Strongyloides* spp.
Takeuchi-Storm et al. (2015)	Denmark	*Felis catus*	Pet and stray cats	GI	AWM	NA	99	1	1.0%	*Strongyloides* spp.
Campos et al. (2016)	Brazil	*Felis catus*	Pet cats	Faeces	FES, FF	NA	160	15	9.4%	*Strongyloides* spp.
Monteiro et al. (2016)	Brazil	*Felis catus*	Pet cats	Faeces	FLOTAC	Eggs	173	24	13.9%	*Strongyloides stercoralis*^b^
Wright, Stafford, & Coles (2016)	England	*Felis catus*	Pet cats	Faeces	FLOTAC	NA	131	2	1.5%	*Strongyloides* spp.
El-Seify et al. (2017)	Egypt	*Felis catus*	Stray cats	Faeces	DS, FF	Eggs	170	1	0.6%	*Strongyloides planiceps*^b^
Giannelli et al. (2017)	Bulgaria	*Felis catus*	Pet cats	Faeces	McMaster, BT	NA	120	16	13.3%	*Strongyloides* spp.
Lima et al. (2017)^a^	Brazil	*Felis catus*	Stray cats	Faeces	FLOTAC	NA	37	20	54.1%	*Strongyloides* spp.
Martinković et al. (2017)	Croatia	*Felis silvestris silvestris*	Wild felids	GI	AWM; FF for rectal faeces	NA	34	8	23.5%	*Strongyloides* spp.
Njuguna et al. (2017)	Kenya	*Felis catus*	Pet cats	Faeces	FES, McMaster	Eggs and larvae	103	45	43.7%	*Strongyloides stercoralis*^b^
Pumidonming et al. (2017)	Thailand	*Felis catus*	Pet cats	Faeces	FF, FES	NA	180	0	0.0%	
Raue et al. (2017)	Germany	*Felis catus*	Pet cats	Faeces	BT, FF	NA	903	0	0.0%	
Solórzano-García et al. (2017)	Mexico	*Panthera onca*	Wild felids	Faeces	FF, SS	Eggs	68	9	13.2%	*Strongyloides* spp.
Solórzano-García et al. (2017)	Mexico	*Puma concolor*	Wild felids	Faeces	FF, SS	Eggs	33	8	23.7%	*Strongyloides* spp.
Solórzano-García et al. (2017)	Mexico	*Unidentified large felids*	Wild felids	Faeces	FF, SS	Eggs	66	12	18.2%	*Strongyloides* spp.
Kostopoulou, et al. (2017)	Greece	*Felis catus*	Pet and stray cats	Faeces	FES, FF	NA	264	0	0.0%	
Iliev et al. (2017)	Bulgaria	*Felis catus*	Pet cats	Faeces	DS, FF	NA	143	4	2.8%	*Strongyloides* spp.
Islam et al. (2018)	Bangladesh	*Felis catus*	Pet cats	Faeces	DS, FES	NA	579	89	15.4%	*Strongyloides* spp.
Sauda et al. (2019)	Italy	*Felis catus*	Shelter cats	Faeces	BT, FF	Larvae	132	1	0.8%	*Strongyloides* spp.
Jitsamai (2019)	Thailand	*Felis catus*	Pet cats	Faeces	FES; APC	Larvae	835	14	1.7%	*Strongyloides felis*
Kurnosova et al. (2019)	Russia	*Felis catus*	Pet cats	Faeces	DS, FF, FES	NA	1261	0	0.0%	
Ko et al. (2020)	Myanmar	*Felis catus*	Shleter cats	Faeces	APC, PCR^c^	Larvae	192	19	9.9%	*Strongyloides* spp.
Ramos et al. (2020)	Brazil	*Felis catus*	Pet cats	Faeces	BT, FF	NA	57	3	5.3%	*Strongyloides* spp.
Ramos et al. (2020)	Brazil	*Felis catus*	Shelter cats	Faeces	BT, FF	NA	336	0	0.0%	
Genchi et al. (2021)	Italy	*Felis catus*	Pet cats	Faeces	Mini-FLOTAC; BT	NA	987	1	0.1%	*Strongyloides stercoralis*^b^
Abbas et al. (2022)	Egypt	*Felis catus*	Stray cats	Faeces	FF	Eggs	143	3	2.1%	*Strongyloides* spp.
Bourgoin et al. (2022)	France	*Felis catus*	Pet cats	Faeces	McMaster	NA	425	0	0.0%	
Colombo et al. (2022)	Italy	*Felis catus*	Pet cats	Faeces	FF, Mini-FLOTAC, BT	NA	105	1	0.9%	*Strongyloides stercoralis*^b^
Henry et al. (2022)	France	*Felis catus*	Pet cats	Faeces	BT, FF	Larvae	448	2	0.4%	*Strongyloides* spp.
Henry et al. (2022)	France	*Felis catus*	Pet cats	GI	AWM, BT for rectal faeces	NA	50	1	2.0%	*Strongyloides* spp.
Adhikari et al. (2023)	Nepal	*Felis catus*	Pet and stray cats	Faeces	DS, FES, FF	Eggs	107	7	6.5%	*Strongyloides* spp.
Mateo et al. (2023)	Spain	*Felis catus*	Pet cats	Faeces	BT, FES	NA	35	0	0.0%	

a Studies excluded from quantitative analysis.

b Methods for species confirmation were unspecified.

c Only samples positive by APC were confirmed by PCR and partial *18S rRNA* sequencing. DS, direct smear; FF, faecal flotation; FES, formalin-ether/ethyl acetate sedimentation; BT, Baermann technique; SS, spontaneous sedimentation; AWM, adult worm morphology; PCR, polymerase chain reaction; GI, gastrointestinal contents and mucosa by necropsy; NA, not applicable.

**Fig. 2:**
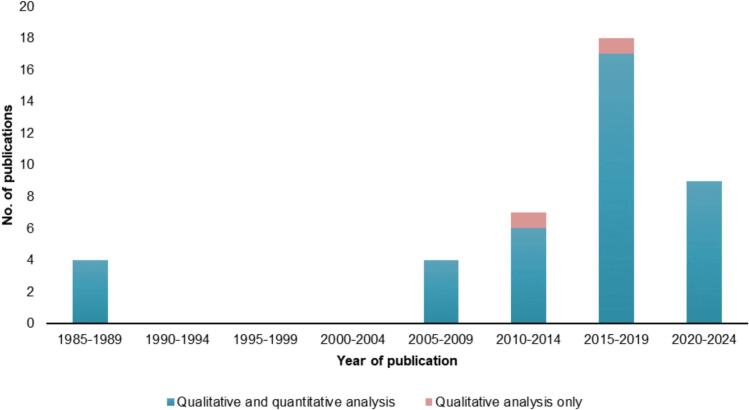
Studies included in qualitative and quantitative analysis by publication year.

Most of the studies were published in the 2010s (60%; 25/42) and 2020s (21%; 9/42) (Fig. 2). Although publications from 1983 to 2024 were eligible for inclusion, no studies were identified prior to 1985 and during 1989–2005.

**Fig. 3:**
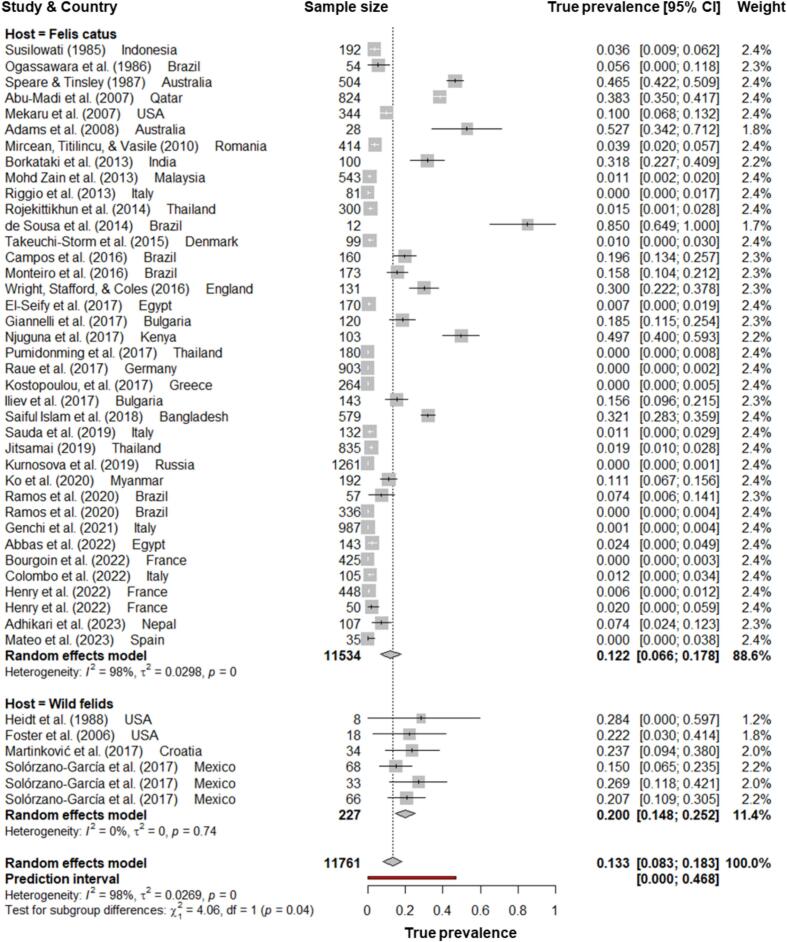
Forest plot of pooled *Strongyloides* prevalence in domestic cats and wild felids.

**Fig. 4:**
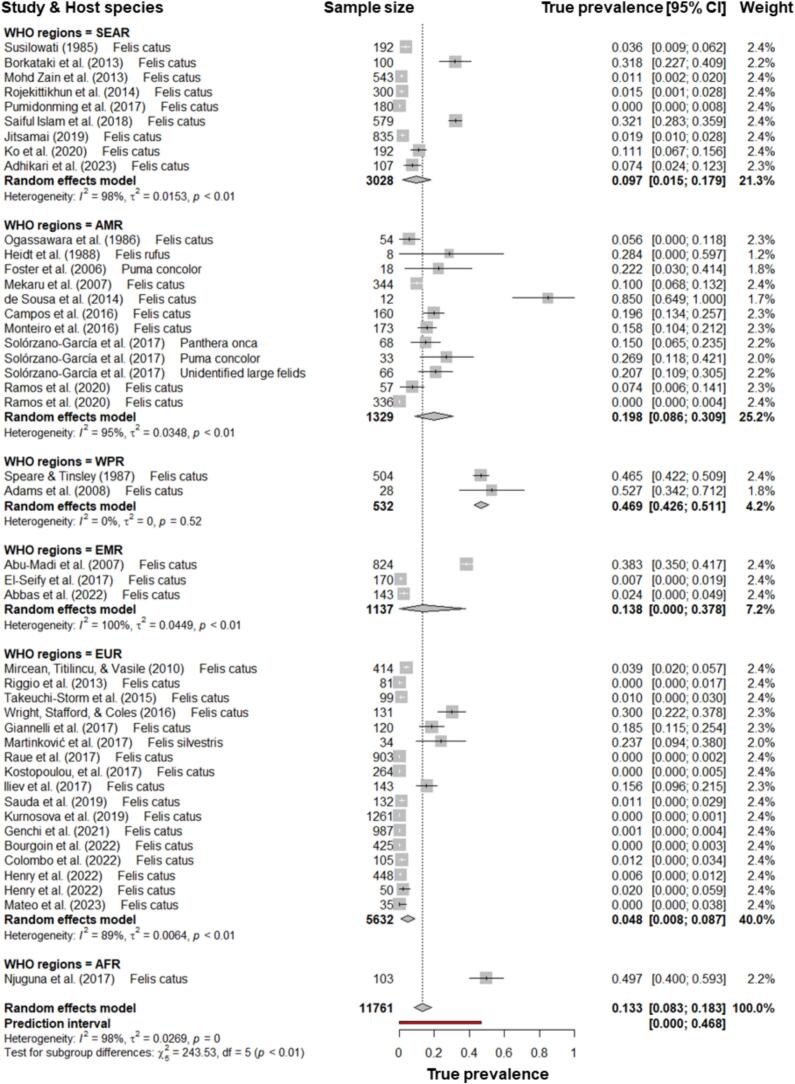
Forest plot of pooled *Strongyloides* prevalence in felines globally and by World Health Organisation regions. SEAR, South-East Asian Region; AMR, American Region; WPR, Western Pacific Region; EMR, Eastern Mediterranean Region; EUR, European Region; AFR, African Region.

The quantitative meta-analysis encompassed 11,761 felines (11,534 domestic cats and 227 wild felids) from 21 countries across six WHO regions. Wild feline host species included *Felis rufus* (*n* = 8) [42], *Puma concolor* (*n* = 51) [43,44], *Felis silvestris* (*n* = 34) [45], *Panthera onca* (*n* = 68) [44], and unidentified large felids (*n* = 66) [44]. Study sample sizes ranged from 8 to 1261, with a median size of 143 (Fig. 3, Fig. 4).

Regarding the diagnostic approach, 86% (38/44) of the studies/datasets relied on parasitological analysis of host faecal samples, while the remaining 14% (6/44) used intestinal adult worm recovery by necropsy. Coproscopic diagnostic techniques, including direct smear (16%; 6/38), faecal flotation (63%; 24/38), spontaneous sedimentation (16%; 6/38), formalin-ether/ethyl acetate sedimentation (29%; 11/38), FLOTAC/mini-FLOTAC (11%; 4/38), McMaster (11%; 4/38), Baermann technique (29%; 11/38), and Agar Plate Culture (APC) (5%; 2/38), were employed either independently or in combination for faecal *Strongyloides* detection. One study [17] utilised PCR and partial *18S rRNA* sequencing, but only samples positive by APC were molecularly confirmed for *Strongyloides*. No study employed serological methods for nematode diagnosis.

Nine studies identified *Strongyloides* to the species level, including *S. stercoralis* in six studies [[46], [47], [48], [49], [50], [51]], *S. felis* in two studies [16,52], and *S. planiceps* in one study [53]. However, 78% (7/9) of these studies lacked details on how species was confirmed. Only two studies, both reporting *S. felis* [16,52], provided morphological evidence for species identification. Oviparous *Strongyloides* spp. were reported in 13 studies, including nine documenting the parasite in domestic cats.

#### 3.1.2 Global prevalence of *Strongyloides* in domestic cats and wild felids

Based on the random effects model, the estimated global pooled prevalence of feline strongyloidiasis was 13.3% (95% CI: 8.3–18.3%) (Fig. 3). The Cochran Q test (*Q* = 1840.32; *df* = 43; *P* < 0.0001) and *I*^*2*^ index (97.7%) indicated a high level of heterogeneity among the studies. The pooled global prevalence of *Strongyloides* spp. in domestic cats (*F. catus*) was 12.2% (95% CI: 6.7–17.8%), considerably lower than that in wild felids (20.0%; 95% CI: 14.9–25.2%) (Table 4).

**Table 4:** Subgroup analyses of *Strongyloides* prevalence in felines.

Subgroups	Number of studies/datasets	TP (%) [95% CI]	*χ*^2^	*p value* *for χ*^2^	*I*^2^
**WHO regions**			243.53	<0.01	
American region	12	19.8 [8.6–30.9]	228.88	<0.01	95.2%
European region	17	4.8 [0.8–8.7]	144.35	<0.01	88.9%
Western Pacific region	2	46.9 [42.6–51.1]	0.41	0.5220	0%
South-East Asian region	9	9.7 [1.5–17.9]	332.36	<0.01	97.6%
Eastern Mediterranean region	3	13.8 [0–37.8]	438.12	<0.01	99.5%
African region	1	49.7 [40.0–59.3]	NA	NA	NA
**Host species**			6.67	0.2465	
*Felis catus*	38	12.2 [6.7–17.8]	1780.91	<0.01	97.9%
*Felis rufus*	1	28.4 [0–59.7]	NA	NA	NA
*Puma concolor*	2	25.1 [13.2–37.0]	0.14	0.7083	0%
*Felis silvestris*	1	23.7 [9.4–38.0]	NA	NA	NA
*Panthera onca*	1	15.0 [6.5–23.5]	NA	NA	NA
Unknown feline species	1	20.7 [10.9–30.4]	NA	NA	NA
**Host types**			36.80	<0.01	
Pet cats	21	9.3 [3.7–14.9]	599.07	<0.01	96.7%
Stray cats	7	29.2 [6.3–52.1]	596.01	<0.01	99.0%
Mixed pet and stray cats	4	13.7 [0–35.3]	439.39	<0.01	99.3%
Shelter cats	5	4.4 [0–9.0]	64.14	<0.01	93.8%
Wild felids	6	20.0 [14.9–25.2]	2.75	0.0973	0%
**Specimen**			19.50	<0.01	
Faeces	38	14.2 [8.5–19.8]	1815.53	<0.01	98.0%
GI contents and mucosa	6	1.3 [0.5–2.1]	16.30	<0.01	69.3%
***Strongyloides* species**			22.94	<0.01	
*Strongyloides stercoralis*	6	18.9 [2.8–35.0]	672.95	<0.01	99.3%
*Strongyloides felis*	2	24.2 [0–67.9]	385.74	<0.01	99.7%
*Strongyloides planiceps*	1	0.7 [0–1.9]	NA	NA	NA
*Strongyloides* spp.	35	11.8 [6.7–16.9]	708.41	<0.01	95.2%

Abbreviation: WHO, World Health Organisation; TP, True prevalence; CI, Confidence Interval; NA, not applicable.

Further analysis based on the host type indicated that stray domestic cats (29.2%; 95% CI: 6.3–52.1%) had the highest pooled prevalence of *Strongyloides* infection among all *F. catus* groups, while shelter domestic cats had the lowest (4.4%; 95% CI: 0–9.0%). Prevalence rates determined using host faecal samples (14.2%; 95% CI: 8.5–19.8%) were significantly higher than those obtained through detection of intestinal adult worms (1.3%; 95% CI: 0.5–2.1%) (*χ*^*2*^ = 19.50; *p* < 0.01). Although only reported by two studies (combined sample sizes: 1339), *S. felis* had the highest prevalence (24.2%; 95% CI: 0–67.9%) among *Strongyloides* spp. in felines (Table 4).

#### 3.1.3 Global distribution of *Strongyloides* in felines

**Fig. 5:**
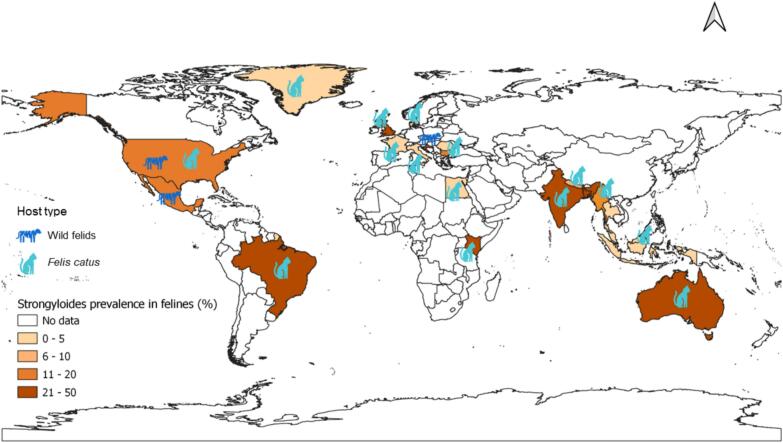
Global prevalence and distribution of *Strongyloides* in felines.

Feline strongyloidiasis were identified across 21 countries in six WHO regions, with the highest pooled prevalence observed in Africa (49.7%; 95% CI: 40.0–59.3%), followed by the Western Pacific (46.9%; 95% CI: 42.6–51.1%). Pooled prevalence was lowest in Europe (4.8%; 95% CI: 0.8–8.7%), followed by South-East Asia (9.7%; 95% CI: 1.5–17.9%) (Fig. 4, Fig. 5).

### 3.2 Cross-species transmission of feline *Strongyloides* species

Seven studies, published between 1925 and 1985, were included in this review (Table 5). Human-to-cat [[54], [55], [56], [57]] or dog-to-cat [58,59] experimental transmission of *S. stercoralis* was described in six studies conducted in the Americas. While in some experiments, cats were refractory to human or canine strains, in most cases, patent short-lived infections lasting 1–7 weeks were established. Additionally, one study described experimental transmission of *S. planiceps* from wild canids and weasels to cats [21]. While this study demonstrated that wild carnivores could potentially serve as reservoirs for *S. planiceps* infection in cats, the potential for this transmission to occur naturally and cats' ability to sustain the infection were not explored.

**Table 5:** A summary of human-cat or dog-cat cross-species transmission studies from the literature.

Year	Geographical origin of infection	*Strongyloides* species	Origin host	Passage host	Recipient host/s (number, age)	no. larvae inoculated and mode of infection	Immunosuppression	Diagnostic method/s	Prepatent period	Patent period	Notes	References
1925	North America (Georgia)	*Strongyloides stercoralis*	Human	Passage through one dog	Cat (n = 3; two adults and one 3 mo old)	iL3 percutaneous, site NS	None	Culture followed by Baermann sedimentation	<13 days	>7 days	Larval passage reached peak at 2–3 days during the patent infection period	[54]
1926	Caribbean (Puerto Rico)	*Strongyloides stercoralis*	Human	Passage through one dog	Cat (*n* = 1, age NS)	12,000 iL3 percutaneous, site NS	None	Charcoal culture followed by Baermann sedimentation	NS	<15 days		[55]
1926	Caribbean (Puerto Rico)	*Strongyloides stercoralis*	Human	NA	Cat (n = 1, age NS)	iL3 percutaneous, site NS	None	Charcoal culture followed by Baermann sedimentation	Refractory	Refractory	Positive faecal culture noted when reinfected later with larvae from the same original human host but passaged through one dog	[55]
1926	Caribbean (Puerto Rico)	*Strongyloides stercoralis*	Human	Passage through two dogs	Cat (n = 1, age NS)	iL3 percutaneous, site NS	None	Charcoal culture followed by Baermann sedimentation	NS	<15 days		[55]
1926	North America (Georgia)	*Strongyloides stercoralis*	Human	NA	Cat (*n* = 9, ‘young and healthy, many were between 2 and 3 years old’)	iL3 percutaneous, site NS	None	Culture (method NS)	NS	1–6 wks	Acute diarrhea in 2/9 of the cats shortly (1–2 days) after the infection, which did not persist or reappear later	[56]
1928	North America (Georgia)	*Strongyloides stercoralis*	Human	Passage through multiple puppies	Cat (n = 1, 4 mo)	12,000 iL3 percutaneous on the abdomen, reinfected with 3800 iL3	None, but poor nutrition owing to poor diet	Charcoal culture followed by Baermann sedimentation	16 days	5–6 wks	No patent infection observed following reinfection (11 cultures performed)	[57]
1928	North America (Georgia)	*Strongyloides stercoralis*	Human	Passage through multiple puppies	Cat (n = 1, >24 mo)	500 iL3 percutaneous on the abdomen, reinfected with 1500 iL3	None, but poor nutrition owing to poor diet	Charcoal culture followed by Baermann sedimentation	15 days	1.5 wks	No patent infection observed following reinfection	[57]
1928	North America (Georgia)	*Strongyloides stercoralis*	Human	Passage through multiple puppies	Cat (n = 1, 6 mo)	1400 iL3 percutaneous on the abdomen	None, but poor nutrition owing to poor diet	Charcoal culture followed by Baermann sedimentation	12 days	2–3 wks		[57]
1928	North America (Georgia)	*Strongyloides stercoralis*	Human	Passage through multiple puppies	Cat (n = 1, >24 mo)	3000 iL3 percutaneous on the abdomen, reinfected twice, with 15,000 iL3 and 26,000 iL3	None, but poor nutrition owing to poor diet	Charcoal culture followed by Baermann sedimentation	9 days	3 wks	No patent infection observed following reinfection	[57]
1928	North America (Georgia)	*Strongyloides stercoralis*	Human	Passage through multiple puppies	Cat (n = 1, >24 mo)	40,000 iL3 percutaneous on the abdomen, reinfected with 10,000 iL3	None, but poor nutrition owing to poor diet	Charcoal culture followed by Baermann sedimentation	14 days	∼1 wk	No patent infection observed following reinfection	[57]
1928	North America (Georgia)	*Strongyloides stercoralis*	Human	Passage through multiple puppies	Cat (n = 1, 18 mo)	10,000 iL3 percutaneous on the abdomen	None, but poor nutrition owing to poor diet	Charcoal culture followed by Baermann sedimentation	NS	2–3 wks	Light infection with diarrhea	[57]
1928	North America (Georgia)	*Strongyloides stercoralis*	Human	Passage through multiple puppies	Cat (n = 1, >12 mo)	2000 iL3 percutaneous on the abdomen, reinfected with 1500 iL3	None, but poor nutrition owing to poor diet	Charcoal culture followed by Baermann sedimentation	12 days	< 2 wks	No patent infection observed following reinfection	[57]
1928	North America (Georgia)	*Strongyloides stercoralis*	Human	Passage through multiple puppies	Cat (n = 1, 9 mo)	3100 iL3 percutaneous on the abdomen, reinfected with 14,000 iL3, 13,000 iL3, 12,000 iL3	None, but poor nutrition owing to poor diet	Charcoal culture followed by Baermann sedimentation	13 days	6 wks	No patent infection observed following reinfection	[57]
1928	North America (Georgia)	*Strongyloides stercoralis*	Human	Passage through multiple puppies	Cat (n = 1, >60 mo)	10,000 iL3 percutaneous on the abdomen	None, but poor nutrition owing to poor diet	Charcoal culture followed by Baermann sedimentation	12 days	7 wks		[57]
1938	North America (Massachusetts)	*Strongyloides stercoralis*	Dog	NA	Cat (*n* = 2, ‘young cats’)	2000–5000 iL3 percutaneous on shaved or clipped areas	None	NA	Refractory	Refractory		[58]
1968	North America (Oklahoma)	*Strongyloides stercoralis*	Dog	NA	Cat (*n* = 1, 4 mo)	1500 iL3 percutaneous on the shoulder	None	Baermann technique	16 days	NS		[59]
1968	North America (Oklahoma)	*Strongyloides stercoralis*	Dog	NA	Cat (n = 1, 4 mo)	700 iL3 percutaneous on the shoulder	None	Baermann technique	16 days	7 days		[59]
1982–1983	Japan (Niigata)	*Strongyloides planiceps*	Racoon dog	NA	Cat (n = 1, age NS)	1000 iL3 percutaneous	None	Direct smear, saturated salt flotation, formalin-ether sedimentation, Harada and Mori's culture	9 days	NS	Embryonated eggs were passed in faeces	[21]
1982–1983	Japan (Niigata)	*Strongyloides planiceps*	Japanese weasel	NA	Cat (n = 1, age NS)	1000 iL3 percutaneous	None	Direct smear, saturated salt flotation, formalin-ether sedimentation, Harada and Mori's culture	10 days	NS	Embryonated eggs were passed in faeces	[21]

wks: weeks; mo: months; NS: not stated; NA: not applicable.

## 4 Discussion

We present, to our knowledge, the first systematic review and meta-analysis to assess the global prevalence and distribution of feline strongyloidiasis. The pooled *Strongyloides* spp. prevalence in felines (13.3%; 95% CI: 8.3–18.3%) and in domestic cats (12.2%; 95% CI: 6.7–17.8) worldwide was markedly higher than the reported *S. stercoralis* prevalence in humans [9] and canines [10,60]. Several factors may contribute to this disparity. It is worth noting that our study included surveillance data for all feline *Strongyloides* spp. Comparability of prevalence rates among canine, feline, and human hosts may be compromised by the inclusion of oviparous cat *Strongyloides* spp. in the present analysis. While human and canine strongyloidiasis are overwhelmingly attributable to *S. stercoralis* based on the faecal passage of *Strongyloides* rhabditiform larvae [1,2], it is currently uncertain in feline cases whether such larvae represent *S. stercoralis* or *S. felis*. Further advanced morphological, or genotypic, characterisation is required to differentiate between these two species. Additionally, unlike dogs, cats bury their faeces and do not tend to defecate in the open, reducing environmental contamination with feline species of *Strongyloides*. Despite this, infection in cats persists, suggesting the possibility of other yet-to-be identified transmission routes. While transmammary transmission has been proposed as a route for canine *S. stercoralis* infection [61], this remains unexamined for felines. This and other vertical transmission routes of *Strongyloides* in cats could be an avenue for future research.

Methodologically, we employed statistical modelling to account for limitations in the diagnostic data from feline studies, an analytical step lacking in comparable meta-analyses for canine strongyloidiasis [10,60]. While this may lead to improved estimation, the prevalence of feline strongyloidiasis could still be underestimated. One possible reason is that, for larvae-shedding feline *Strongyloides* spp., a negative faecal test may not necessarily reflect the absence of disease due to low and intermittent larval output [34]. Although isolation of intestinal worms by necropsy is deemed more sensitive [34], it was only performed in 14% (6/44) of the studies. Another contributing factor to underestimation is the skewed representation of studies from the American (27%; 12/44) and European (39%; 17/44) regions. The paucity of surveillance data from low-income regions, such as sub-Saharan Africa, where veterinary services are often limited, inaccessible, or unaffordable [62], may bias the assessment of the true global feline disease burden.

Globally, feline strongyloidiasis was not restricted to tropical and subtropical regions, although prevalence was generally higher in these areas, mirroring patterns observed in human [9] and canine [10,60] strongyloidiasis. Lower income WHO regions, such as Africa (49.7%; 95% CI: 40.0–59.3%) and the Western Pacific (46.9%; 95% CI: 42.6–51.1%), had the highest pooled *Strongyloides* spp. prevalence in felines, consistent with findings for the nematode in canines [10]. Geographical variations in prevalence may be attributable to climatic, environmental, and socio-economic factors. Laboratories and veterinarians in regions with high rates of, or high awareness of *Aelurostrongylus abstrusus* infection may be more inclined to perform larval recovery methods such as the Baermann technique, incidentally also identifying more infections with larviparous *Strongyloides* spp. The wide confidence intervals of prevalence rates for some WHO regions, such as the Eastern Mediterranean region (13.8%; 0–37.8%) and South-East Asian region (9.7%; 1.5–17.9%), indicate considerable uncertainty in the estimated prevalence. Future surveillance studies with large sample sizes from diverse geographical areas are warranted.

The high level of heterogeneity (*I*^*2*^ = 97.7%; *Q* = 1840.32; *df* = 43; *P* < 0.0001) observed among the studies in this review prompted a further investigation into host-specific factors influencing the epidemiology of feline strongyloidiasis. In line with findings for other soil-transmitted helminths in felines [[63], [64], [65]], stray domestic cats (*F. catus*) had a significantly higher prevalence of *Strongyloides* spp. compared to their owned counterparts (pet and shelter cats) in more controlled environments. This difference may be explained by the unrestricted outdoor activities of stray cats, potentially increasing environmental transmission of the parasite, coupled with the absence of anthelmintic therapy or other veterinary care for this population.

This review found that *Strongyloides* detection in feline surveys predominantly relied on traditional microscopy, with flotation-based methods being the most commonly employed diagnostic approach. Faecal flotation methods are ineffective for *Strongyloides* larvae recovery [4,34] and thus are unreliable for detecting *S. stercoralis*, *S. felis,* and *S. tumefaciens* in feline faeces. Its sensitivity for faecal *S. planiceps* egg detection remains to be tested. While APC is recommended for isolating the larviparous *Strongyloides* spp. and potentially allows species differentiation by morphologists [34], its utilisation in existing feline studies was very limited (5%; 2/38).

Only two studies in this review provided robust morphological or molecular evidence for species-level *Strongyloides* identification [16,52]. This makes it challenging to examine the prevalence of individual *Strongyloides* spp. in felines. Given recent phylogenetic evidence suggesting the zoonotic potential of certain cat *S. stercoralis* strains [23], surveillance of this species and genotypes in felines using highly sensitive molecular tools is necessary from a public health perspective. Egg-shedding *Strongyloides* spp., potentially representing *S. planiceps*, were reported in 13 studies, in both wild felids and domestic cats. While experimentally demonstrated [21], *S. planiceps*'s capacity to cause natural infection in *F. catus* and its veterinary impact remain unclear and could benefit from future research.

Historical cross-infection studies revealed that cats were poorly susceptible to human- or canine-derived *S. stercoralis*. Intensity of larval inoculation did not have any detectable influence on subsequent duration of infection. In Sandground [55]’s series of experiments, the passage of human-derived *S. stercoralis* through dogs seemed to enhance its cross-infectivity in cats. However, in other studies, direct inoculation with dog strains either failed to induce infection [58] or only resulted in transient infections lasting seven days in cats [59]. Interpretation of these findings require caution, as these experiments were conducted before molecular genotyping was available, so results may be confounded by potentially differing felid infectivity of different genotypes. Additionally, variations in inoculation procedures and diagnostic approaches may limit direct comparability between experiments or to natural infections, and there was a sampling bias towards strains originating in North America. Furthermore, studies in dogs have identified that *S. stercoralis* parasitic female can enter a barren phase in which fertile eggs are not produced, but fecundity may return later under specific conditions [66]. Therefore, without investigation by necropsy, it cannot be definitively determined that the absence of larval shedding after a few weeks of infection reflects true host clearance of the infection, or entry of the parasitic females into a senescent phase. The data from existing cross-infection studies suggests that cats are relatively refractory to infection with *S. stercoralis* from dogs or humans and may not be a significant reservoir for natural *S. stercoralis* infections in those hosts, but this evidence remains limited and inconclusive.

The meta-analysis has several limitations. Firstly, the sensitivity and specificity data used for TP calculation were mostly derived from studies of human, dog, or non-human primate hosts, which may not be generalisable to feline hosts due to potential variations in hosts' faecal composition. Moreover, these diagnostic performance data are imperfect owning to inconsistent reference standards used across studies. Consequently, the accuracy of the pooled prevalence may be compromised. Secondly, most of the included studies focused on general intestinal parasitism in felines, without specifically targeting *Strongyloides*. Faecal *Strongyloides* detection is challenging and requires experienced morphologists, as the larvae demonstrate low and irregular output, making them easily overlooked, while the eggs can be mistaken for those of hookworms. This may lead to potential underestimation of the pooled prevalence. Thirdly, there is a paucity of country-level data, impeding an unbiased assessment of feline strongyloidiasis prevalence in different WHO regions. Notably, the pooled prevalence for the African region relied on data collected in a single country (Kenya), with a sample size of 103 [49].

## 5 Conclusion

This systematic review and meta-analysis highlights the importance of ongoing research, control, and surveillance for feline strongyloidiasis globally. The continued reliance on inadequate diagnostic approaches in most feline surveys remains a challenge for evaluating the true disease burden. Furthermore, the role of cross-species transmission in feline, human and canine *S. stercoralis* infection is not fully understood. To determine whether cats are truly a significant factor in the epidemiology of human or canine strongyloidiasis, molecular taxonomy studies and controlled population treatment experiments may be beneficial. This could involve sampling cats, dogs, and humans from the same communities and comparing population genetics of *S. stercoralis* from these hosts. Exploring the impact of co-treatment of cats on the infection dynamics in different hosts may also provide insights into the role cats might or might not play in human and canine infections.

## Funding

The first author receives an Australian Government Research Training Program Scholarship from James Cook University. The funder had no role in study design, data collection and analysis, decision to publish, or preparation of the manuscript.

## CRediT authorship contribution statement

**Huan Zhao:** Conceptualization, Data curation, Formal analysis, Methodology, Writing – original draft. **Richard Stewart Bradbury:** Conceptualization, Data curation, Funding acquisition, Resources, Supervision, Writing – review & editing.

## Declaration of competing interest

None.

## Data Availability

Data will be made available on request.

## Appendix A Supplementary data

Supplementary material 1 Quality assessment of included studies for risk of bias using the JBI critical appraisal tool Supplementary material 1

Supplementary material 2 References for included studies in the systematic review Supplementary material 2
