# Supplementary material for: Feline strongyloidiasis: An insight into its global prevalence and transmission cycle
Source: One Health. 2024 Jun 20;19:100842. doi: 10.1016/j.onehlt.2024.100842 (PMC11255105; doi:10.1016/j.onehlt.2024.100842)
Supplement: Supplementary material 1 — Quality assessment of included studies for risk of bias using the JBI critical appraisal tool [file mmc1.docx]

**Table S1**. Quality assessment of included studies for risk of bias using the JBI critical appraisal tool

| **Authors (year)** | **Was the sample frame appropriate to address the target population?** | **Were study participants sampled in an appropriate way?** | **Was the sample size adequate?** | **Were the study subjects and the setting described in detail?** | **Was the data analysis conducted with sufficient coverage of the identified sample?** | **Were valid methods used for the identification of the condition?** | **Was the condition measured in a standard, reliable way for all participants?** | **Was there appropriate statistical analysis?** | **Score** |
| --- | --- | --- | --- | --- | --- | --- | --- | --- | --- |
| Susilowati (1985) | Y | Y | Y | N | Y | Y | N | Y | 6 |
| Ogassawara et al. (1986) | Y | Y | N | Y | Y | Y | N | Y | 6 |
| Speare & Tinsley (1987) | Y | Y | Y | Y | Y | Y | Y | Y | 8 |
| Heidt et al. (1988) | Y | Y | N | Y | Y | N | N | U | 4 |
| Foster et al. (2006) | Y | Y | N | Y | Y | Y | Y | Y | 7 |
| Abu-Madi et al. (2007) | Y | Y | Y | Y | Y | Y | Y | Y | 8 |
| Mekaru et al. (2007) | Y | Y | Y | Y | Y | N | N | Y | 6 |
| Adams et al. (2008) | Y | Y | N | Y | Y | N | N | Y | 5 |
| Mircean, Titilincu, & Vasile (2010) | Y | Y | Y | Y | Y | N | N | Y | 6 |
| Borkataki et al. (2013) | Y | Y | Y | Y | Y | Y | N | Y | 7 |
| Mohd Zain et al. (2013) | Y | Y | Y | Y | Y | Y | Y | Y | 8 |
| Aranda R. et al. (2013)* | N | N | N | Y | Y | Y | Y | U | 4 |
| Riggio et al. (2013) | Y | Y | Y | Y | Y | Y | N | Y | 7 |
| Rojekittikhun et al. (2014) | Y | Y | Y | Y | Y | Y | Y | Y | 8 |
| de Sousa et al. (2014) | Y | Y | N | Y | Y | Y | N | Y | 6 |
| Takeuchi-Storm et al. (2015) | Y | Y | Y | Y | Y | Y | Y | Y | 8 |
| Campos et al. (2016) | Y | Y | Y | Y | Y | Y | N | Y | 7 |
| Monteiro et al. (2016) | Y | Y | Y | N | Y | N | N | Y | 5 |
| Wright, Stafford, & Coles (2016) | Y | Y | Y | N | Y | N | N | Y | 5 |
| El-Seify et al. (2017) | Y | Y | Y | Y | Y | Y | N | Y | 7 |
| Giannelli et al. (2017) | Y | Y | Y | Y | Y | Y | N | Y | 7 |
| Lima et al. (2017)* | Y | U | N | N | Y | N | N | U | 2 |
| Martinković et al. (2017) | Y | Y | N | Y | Y | Y | Y | Y | 7 |
| Njuguna et al. (2017) | Y | Y | Y | Y | Y | Y | N | Y | 7 |
| Pumidonming et al. (2017) | Y | Y | Y | Y | Y | Y | N | Y | 7 |
| Raue et al. (2017) | Y | Y | Y | Y | Y | Y | N | Y | 7 |
| Solórzano-García et al. (2017) | N | Y | N | Y | Y | Y | Y | U | 5 |
| Kostopoulou et al. (2017) | Y | Y | Y | Y | Y | Y | N | Y | 7 |
| Iliev et al. (2017) | Y | Y | Y | Y | Y | Y | N | Y | 7 |
| Saiful Islam et al. (2018) | Y | Y | Y | Y | Y | Y | N | Y | 7 |
| Sauda et al. (2019) | Y | Y | Y | Y | Y | Y | Y | Y | 8 |
| Jitsamai (2019) | Y | Y | Y | Y | Y | Y | Y | Y | 8 |
| Kurnosova et al. (2019) | Y | Y | Y | Y | Y | Y | N | Y | 7 |
| Ko et al. (2020) | Y | Y | Y | Y | Y | Y | Y | Y | 8 |
| Ramos et al. (2020) | Y | Y | N | Y | Y | Y | N | Y | 6 |
| Genchi et al. (2021) | Y | Y | Y | Y | Y | Y | N | Y | 7 |
| Abbas et al. (2022) | Y | Y | Y | Y | Y | N | N | Y | 6 |
| Bourgoin et al. (2022) | Y | Y | Y | Y | Y | Y | N | Y | 7 |
| Colombo et al. (2022) | Y | Y | Y | Y | Y | Y | N | Y | 7 |
| Henry et al. (2022) | Y | Y | N | Y | Y | Y | Y | Y | 7 |
| Adhikari et al. (2023) | U | Y | Y | Y | Y | Y | N | Y | 6 |
| Mateo et al. (2023) | Y | Y | N | Y | Y | Y | N | Y | 6 |

*, Studies indicating a high risk of bias; Abbreviations: Y, yes; N, no; U, unclear; N/A, not applicable
