# Supplementary material for: Feline strongyloidiasis: An insight into its global prevalence and transmission cycle
Source: One Health. 2024 Jun 20;19:100842. doi: 10.1016/j.onehlt.2024.100842 (PMC11255105; doi:10.1016/j.onehlt.2024.100842)
Supplement: Supplementary material 2 — References for included studies in the systematic review [file mmc2.docx]

**References for included studies**

[1] Abbas, I., Al-Araby, M., Elmishmishy, B. and El-Alfy, E. S. Gastrointestinal parasites of cats in Egypt: high prevalence high zoonotic risk. *BMC Vet Res*, 18, 1 (Nov 29 2022), 420.

[2] Abu-Madi, M., Al-Ahbabi, D., Al-Mashhadani, M., Al-Ibrahim, R., Pal, P. and Lewis, J. Patterns of parasitic infections in faecal samples from stray cat populations in Qatar. *Journal of helminthology*, 81, 3 (2007), 281-286.

[3] Adams, P., Elliot, A., Algar, D. and Brazell, R. Gastrointestinal parasites of feral cats from Christmas Island. *Australian veterinary journal*, 86, 1‐2 (2008), 60-63.

[4] Adhikari, R. B., Dhakal, M. A., Ale, P. B., Regmi, G. R. and Ghimire, T. R. Survey on the prevalence of intestinal parasites in domestic cats (Felis catus Linnaeus, 1758) in central Nepal. *Vet Med Sci*, 9, 2 (Mar 2023), 559-571.

[5] Aranda, C., Serrano-Martínez, E., Tantaleán, M., Quispe, M. and Casas, G. Identificación y frecuencia de parásitos gastrointestinales en félidos silvestres en cautiverio en el Perú. *Revista de Investigaciones Veterinarias del Perú*, 24, 3 (2013), 360-368.

[6] Borkataki, S., Katoch, R., Goswami, P., Godara, R., Khajuria, J., Yadav, A. and Kaur, R. Prevalence of parasitic infections of stray cats in Jammu, India. *Sokoto Journal of Veterinary Sciences*, 11, 1 (2013), 1-6.

[7] Bourgoin, G., Callait-Cardinal, M. P., Bouhsira, E., Polack, B., Bourdeau, P., Roussel Ariza, C., Carassou, L., Lienard, E. and Drake, J. Prevalence of major digestive and respiratory helminths in dogs and cats in France: results of a multicenter study. *Parasit Vectors*, 15, 1 (Sep 6 2022), 314.

[8] Campos, D. R., Oliveira, L. C., de Siqueira, D. F., Perin, L. R., Campos, N. C., Aptekmann, K. P. and Martins, I. V. Prevalence and risk factors associated with endoparasitosis of dogs and cats in Espírito Santo, Brazil. *Acta Parasitol*, 61, 3 (Sep 1 2016), 544-548.

[9] Colombo, M., Morelli, S., Damiani, D., Del Negro, M. A., Milillo, P., Simonato, G., Barlaam, A. and Di Cesare, A. Comparison of Different Copromicroscopic Techniques in the Diagnosis of Intestinal and Respiratory Parasites of Naturally Infected Dogs and Cats. *Animals*, 12, 19 (2022), 2584.

[10] de Sousa¹, T. N., de Sousa¹, A. C. B., dos Santos Sousa¹, D. G. and Freire, S. M. Ocorrência de parasitos gastrintestinais de gatos (Felis catus) que frequentam a Universidade Estadual do Piauí, Campus Torquato Neto, Teresina (PI) Occurrence of gastrointestinal parasites of cats (Felis catus) attending the State University of Piauí, Ca. *Pubvet*, 8 (2015), 2806-2887.

[11] El-Seify, M. A., Aggour, M. G., Sultan, K. and Marey, N. M. Gastrointestinal helminths of stray cats in Alexandria, Egypt: A fecal examination survey study. *Veterinary Parasitology: Regional Studies and Reports*, 8 (2017), 104-106.

[12] Foster, G. W., Cunningham, M. W., Kinsella, J. M., McLaughlin, G. and Forrester, D. J. Gastrointestinal helminths of free-ranging Florida panthers (Puma concolor coryi) and the efficacy of the current anthelmintic treatment protocol. *Journal of wildlife diseases*, 42, 2 (2006), 402-406.

[13] Genchi, M., Vismarra, A., Zanet, S., Morelli, S., Galuppi, R., Cringoli, G., Lia, R., Diaferia, M., Frangipane di Regalbono, A. and Venegoni, G. Prevalence and risk factors associated with cat parasites in Italy: a multicenter study. *Parasites & Vectors*, 14 (2021), 1-11.

[14] Giannelli, A., Capelli, G., Joachim, A., Hinney, B., Losson, B., Kirkova, Z., René-Martellet, M., Papadopoulos, E., Farkas, R., Napoli, E., Brianti, E., Tamponi, C., Varcasia, A., Margarida Alho, A., Madeira de Carvalho, L., Cardoso, L., Maia, C., Mircean, V., Mihalca, A. D., Miró, G., Schnyder, M., Cantacessi, C., Colella, V., Cavalera, M. A., Latrofa, M. S., Annoscia, G., Knaus, M., Halos, L., Beugnet, F. and Otranto, D. Lungworms and gastrointestinal parasites of domestic cats: a European perspective. *Int J Parasitol*, 47, 9 (Aug 2017), 517-528.

[15] Heidt, G. A., Rucker, R. A., Kennedy, M. L. and Baeyens, M. E. Hematology, intestinal parasites, and selected disease antibodies from a population of bobcats (Felis rufus) in central Arkansas. *Journal of Wildlife diseases*, 24, 1 (1988), 180-183.

[16] Henry, P., Huck-Gendre, C., Franc, M., Williams, T. L., Bouhsira, E. and Lienard, E. Epidemiological Survey on Gastrointestinal and Pulmonary Parasites in Cats Around Toulouse (France). *Helminthologia*, 59, 4 (Dec 2022), 385-397.

[17] Iliev, P., Kirkova, Z., Ivanov, A., Prelezov, P., Tonev, A. and Kalkanov, I. Retrospective analysis on helminthic and protozoan infections in dogs and cats in Bulgaria. *Bulg. J. Vet. Med*, 20, Suppl 1 (2017), 389-393.

[18] Islam, K. S., Shiraj-Um-Mahmuda, S., Kabir, M. H. B. and Sarkar, S. K. Owner perception, zoonotic potential and public health significance of intestinal parasitism in pet cats of Bangladesh. *Journal of advancement in Medical and life sciences*, 7, 1 (2018), 2348-2294X.

[19] Jitsamai, W. Prevalence of enteric helminths and protozoa and identification of hookworm, threadworm and giardia spp. In cats in Bangkok and vicinity, Thailand (2019).

[20] Ko, P. P., Suzuki, K., Canales-Ramos, M., Aung, M., Htike, W. W., Yoshida, A., Montes, M., Morishita, K., Gotuzzo, E., Maruyama, H. and Nagayasu, E. Phylogenetic relationships of Strongyloides species in carnivore hosts. *Parasitol Int*, 78 (Oct 2020), 102151.

[21] Kostopoulou, D., Claerebout, E., Arvanitis, D., Ligda, P., Voutzourakis, N., Casaert, S. and Sotiraki, S. Abundance, zoonotic potential and risk factors of intestinal parasitism amongst dog and cat populations: The scenario of Crete, Greece. *Parasit Vectors*, 10, 1 (Jan 25 2017), 43.

[22] Kurnosova, O. P., Arisov, M. V. and Odoyevskaya, I. M. Intestinal Parasites of Pets and Other House-kept Animals in Moscow. *Helminthologia*, 56, 2 (Jun 2019), 108-117.

[23] Lima, V. F. S., Ramos, R. A. N., Lepold, R., Borges, J. C. G., Ferreira, C. D., Rinaldi, L., Cringoli, G. and Alves, L. C. Gastrointestinal parasites in feral cats and rodents from the Fernando de Noronha Archipelago, Brazil. *Revista Brasileira de Parasitologia Veterinária*, 26 (2017), 521-524.

[24] Martinković, F., Sindičić, M., Lučinger, S., Štimac, I., Bujanić, M., Živičnjak, T., Stojčević Jan, D., Šprem, N., Popović, R. and Konjević, D. Endoparasites of wildcats in Croatia. *Veterinarski arhiv*, 87, 6 (2017), 713-729.

[25] Mateo, M., Montoya, A., Bailo, B., Köster, P. C., Dashti, A., Hernández-Castro, C., Saugar, J. M., Matas, P., Xiao, L. and Carmena, D. Prevalence and public health relevance of enteric parasites in domestic dogs and cats in the region of Madrid (Spain) with an emphasis on Giardia duodenalis and Cryptosporidium sp. *Vet Med Sci*, 9, 6 (Nov 2023), 2542-2558.

[26] Mekaru, S. R., Marks, S. L., Felley, A. J., Chouicha, N. and Kass, P. H. Comparison of direct immunofluorescence, immunoassays, and fecal flotation for detection of Cryptosporidium spp. and Giardia spp. in naturally exposed cats in 4 Northern California animal shelters. *Journal of Veterinary Internal Medicine*, 21, 5 (2007), 959-965.

[27] Mircean, V., Titilincu, A. and Vasile, C. Prevalence of endoparasites in household cat (Felis catus) populations from Transylvania (Romania) and association with risk factors. *Veterinary parasitology*, 171, 1-2 (2010), 163-166.

[28] Mohd Zain, S. N., Sahimin, N., Pal, P. and Lewis, J. W. Macroparasite communities in stray cat populations from urban cities in Peninsular Malaysia. *Vet Parasitol*, 196, 3-4 (Sep 23 2013), 469-477.

[29] Monteiro, M. F. M., Ramos, R. A. N., Calado, A. M. C., Lima, V. F. S., Ramos, I. C. d. N., Tenório, R. F. L., Faustino, M. A. d. G. and Alves, L. C. Gastrointestinal parasites of cats in Brazil: frequency and zoonotic risk. *Revista Brasileira de Parasitologia Veterinária*, 25 (2016), 254-257.

[30] Nyambura Njuguna, A., Kagira, J. M., Muturi Karanja, S., Ngotho, M., Mutharia, L. and Wangari Maina, N. Prevalence of Toxoplasma gondii and Other Gastrointestinal Parasites in Domestic Cats from Households in Thika Region, Kenya. *Biomed Res Int*, 2017 (2017), 7615810.

[31] Ogassawara, S., Benassi, S., Larsson, C. E., Leme, P. T. Z. and Hagiwara, M. K. Prevalência de infecções helmínticas em gatos na cidade de São Paulo. *Revista da Faculdade de Medicina Veterinária e Zootecnia da Universidade de São Paulo*, 23, 2 (1986), 145-149.

[32] Pumidonming, W., Salman, D., Gronsang, D., Abdelbaset, A. E., Sangkaeo, K., Kawazu, S. I. and Igarashi, M. Prevalence of gastrointestinal helminth parasites of zoonotic significance in dogs and cats in lower Northern Thailand. *J Vet Med Sci*, 78, 12 (Jan 10 2017), 1779-1784.

[33] Ramos, N. V., Silva, M. L. E., Barreto, M. S., Barros, L. A. and Mendes-de-Almeida, F. Endoparasites of household and shelter cats in the city of Rio de Janeiro, Brazil. *Rev Bras Parasitol Vet*, 29, 1 (2020), e012819.

[34] Raue, K., Heuer, L., Böhm, C., Wolken, S., Epe, C. and Strube, C. 10-year parasitological examination results (2003 to 2012) of faecal samples from horses, ruminants, pigs, dogs, cats, rabbits and hedgehogs. *Parasitol Res*, 116, 12 (Dec 2017), 3315-3330.

[35] Riggio, F., Mannella, R., Ariti, G. and Perrucci, S. Intestinal and lung parasites in owned dogs and cats from central Italy. *Vet Parasitol*, 193, 1-3 (Mar 31 2013), 78-84.

[36] Rojekittikhun, W., Chaisiri, K., Mahittikorn, A., Pubampen, S., Sa-Nguankiat, S., Kusolsuk, T., Maipanich, W., Udonsom, R. and Mori, H. Gastrointestinal parasites of dogs and cats in a refuge in Nakhon Nayok, Thailand. *Southeast Asian J Trop Med Public Health*, 45, 1 (Jan 2014), 31-39.

[37] Sauda, F., Malandrucco, L., De Liberato, C. and Perrucci, S. Gastrointestinal parasites in shelter cats of central Italy. *Vet Parasitol Reg Stud Reports*, 18 (Dec 2019), 100321.

[38] Solórzano-García, B., White-Day, J. M., Gómez-Contreras, M., Cristóbal-Azkárate, J., Osorio-Sarabia, D. and Rodríguez-Luna, E. Coprological survey of parasites of free-ranging jaguar (Panthera onca) and puma (Puma concolor) inhabiting 2 types of tropical forests in Mexico. *Revista mexicana de biodiversidad*, 88, 1 (2017), 146-153.

[39] Speare, R. and Tinsley, D. Survey of cats for Strongyloides felis. *Aust Vet J*, 64, 6 (Jun 1987), 191-192.

[40] Susilowati, S. *Kejadian Infestasi Cacing Nematoda Dalam Saluran Pencernakan Kucing Di Wilayah Surabaya Utara*. Universias Airlangga, 1985.

[41] Takeuchi-Storm, N., Mejer, H., Al-Sabi, M. N., Olsen, C. S., Thamsborg, S. M. and Enemark, H. L. Gastrointestinal parasites of cats in Denmark assessed by necropsy and concentration McMaster technique. *Vet Parasitol*, 214, 3-4 (Dec 15 2015), 327-332.

[42] Wright, I., Stafford, K. and Coles, G. The prevalence of intestinal nematodes in cats and dogs from Lancashire, north-west England. *J Small Anim Pract*, 57, 8 (Aug 2016), 393-395.
